# Induction of a stable sigma factor SigR by translation-inhibiting antibiotics confers resistance to antibiotics

**DOI:** 10.1038/srep28628

**Published:** 2016-06-27

**Authors:** Ji-Sun Yoo, Gyeong-Seok Oh, Sungweon Ryoo, Jung-Hye Roe

**Affiliations:** 1 Laboratory of Molecular Microbiology, School of Biological Sciences, and Institute of Microbiology, Seoul National University, Seoul 151-742, Korea; 2 Korean Institute of Tuberculosis, 168-5, Osongsaengmyeong 4-ro, Osong, Cheongwon-gun, Chungcheongbuk-do, 28158, Korea

## Abstract

Antibiotic-producing streptomycetes are rich sources of resistance mechanisms against endogenous and exogenous antibiotics. An ECF sigma factor σ^R^ (SigR) is known to govern the thiol-oxidative stress response in *Streptomyces coelicolor*. Amplification of this response is achieved by producing an unstable isoform of σ^R^ called σ^R′^. In this work, we present evidence that antibiotics induce the SigR regulon via a redox-independent pathway, leading to antibiotic resistance. The translation-inhibiting antibiotics enhanced the synthesis of stable σ^R^, eliciting a prolonged response. WblC/WhiB7, a WhiB-like DNA-binding protein, is responsible for inducing *sigR*p1 transcripts encoding the stable σ^R^. The amount of WblC protein and its binding to the *sigR*p1 promoter *in vivo* increased upon antibiotic treatment. A similar phenomenon appears to exist in *Mycobacterium tuberculosis* as well. These findings reveal a novel antibiotic-induced resistance mechanism conserved among actinomycetes, and also give an explicit example of overlap in cellular damage and defense mechanisms between thiol-oxidative and anti- translational stresses.

Many actinomycetes, especially those of *Streptomyces* genus, are well recognized for undergoing complex developmental programs and producing diverse secondary metabolites. In soil environment where streptomycetes inhabit, thousands of bacterial species are estimated to reside in one gram of soil producing more than 10^4^ bioactive small molecules^1^,^2^. In natural environment, streptomycetes have to deal with numerous growth-inhibitory antibiotics which are made by themselves (endogenous) or other organisms (exogenous). Therefore, while being major producers of antibiotics, actinomycetes are the major sources of antibiotic resistance mechanisms^3^. The mechanisms of antibiotic resistance found in clinical pathogens derive their origin from environmental bacteria as first identified for aminoglycoside resistance in *Streptomyces*^4^. Since then, various parallel examples, such as *vanHAX* gene cluster for vancomycin resistance, were reported in soil actinomycetes as well as in clinical strains^5^.

Whether living inside the human body or in natural environment, bacteria are exposed to wide concentration ranges of antibiotics. In most cases, they are exposed to non-lethal or sub-minimal inhibitory concentration (MIC) of antibiotics. Antibiotics at sub-MIC act as signals and stressors to elicit physiological and genetic changes to cope with antibiotic stress^6^,^7^,^8^. Antibiotic resistance phenotype is induced by sub-inhibitory antibiotics through modulating gene expression and physiology (intrinsic resistance) or through changing genetic information via mutation or horizontal transfer of resistance genes (acquired resistance). Modulation of bacterial gene expression to enhance intrinsic resistance is mediated via hosts of regulators. Some known transcriptional regulators include RNA polymerase sigma factors such as RpoS^9^, a redox-sensitive regulator such as SoxR^10^,^11^, or a WhiB-like factor (WblC/WhiB7^12^,^13^). Unraveling the vast array of regulatory pathways and their networks are needed to understand and control resistance mechanisms.

Among regulators that respond to environmental changes, a group of alternative sigma factors called extra-cytoplasmic function (ECF) sigma factors are abundantly encoded in bacterial genomes^14^,^15^. They are also called group 4 sigma factors consisting of only σ_2_ and σ_4_ domains that recognize −35 and −10 regions, respectively, of cognate promoters^16^,^17^. In *Streptomyces coelicolor*, 50 such factors are encoded in the genome^18^. Among them, the role of only several factors has been elucidated, such as SigR (SCO5216^19^), BldN (SCO3323^20^), SigU (SCO2964^21^), SigE (SCO3356^22^), SigT (SCO3892^23^) and SigQ (SCO4908^24^).

The SigR system in *S. coelicolor* is activated by thiol-reactive chemicals that oxidize or alkylate cysteine thiols^19^,^25^. The induction mechanism involves the inactivation of its anti-sigma factor RsrA via forming disulfide bonds, and liberating active SigR^26^,^27^,^28^, which then positively regulates the expression of its own gene from the SigR-dependent upstream promoter (*sigR*p2) (Fig. 1A). The positively amplified *sigR*p2-derived SigR protein contains N-terminally extended 55 more amino acid residues, and is called σ^R′^ to distinguish it from the apparently constitutive form σ^R^ expressed from the downstream promoter (*sigR*p1)^29^. A prominent difference between σ^R^ and σ^R′^ is in their stability. Whereas σ^R^ is stable for hours, σ^R′^ is short-lived with a half-life of ~10 min^29^. Both σ^R^ and σ^R′^ in their free state bind the core RNA polymerase and transcribe over 100 target genes to cope with the thiol-oxidative stress^30^. The SigR regulon includes the thiol-reducing systems which contribute to reactivating RsrA via disulfide reduction. It also includes proteases which degrade σ^R′^, thereby turning off the response within an hour^26^,^29^. Therefore, the response of SigR-RsrA system to thiol-reactive chemical stresses is transient and is mediated by sensor RsrA and amplified σ^R′^.

In this study, we demonstrate that multiple antibiotics induce the SigR system via yet another pathway of signal transduction, different from what conveys the thiol-perturbing signals. We show that the antibiotic induction of the SigR system proceeds via increasing the production of stable σ^R^, and this induction is mediated by WblC/WhiB7. WblC is a WhiB-like protein conserved in actinomycetes^31^,^32^,^33^ and reported to confer resistance to antibiotics in *Mycobacterium* and *Streptomyces*^12^,^34^. WblC/WhiB7 proteins contain three functional domains such as an Fe-S cluster binding domain with four conserved cysteines, a G(V/I)WGG turn, and an AT-hook DNA binding domain^35^. The *whiB7* gene is known to be induced by a variety of antibiotics via autoregulation, and WhiB7 may contribute to intrinsic resistance to antibiotics by activating antibiotic export, antibiotic inactivation and changes in thiol redox balance in mycobacteria^13^,^36^. Our work verifies *sigR*p1 promoter region as a novel binding site of WblC/WhiB7 in *S. coelicolor*, and suggests that the expression of SigR-homologous ECF sigma factor genes (*sigE* and *sigH*) in *M. tuberculosis* may also respond to antibiotics via WhiB7.

## Results

### Induction of the *sigR* gene expression by translation-inhibiting antibiotics

**Figure 1:**
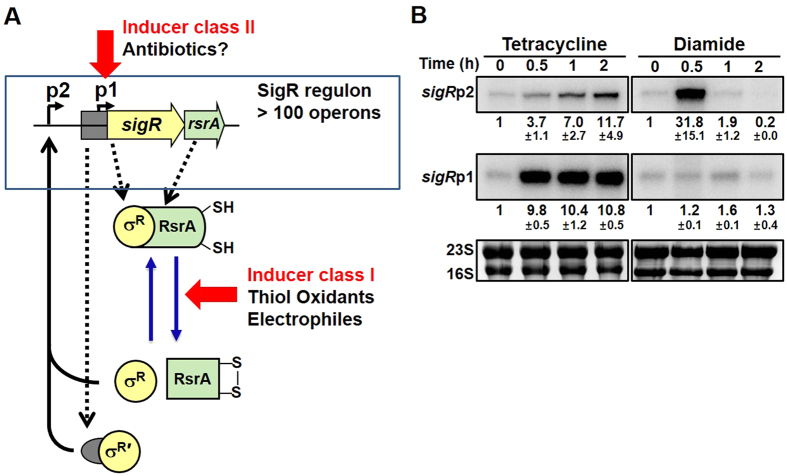
Induction of *sigR* transcription by antibiotics via downstream p1 promoter. (**A**) The regulatory loops in activating SigR regulon. Two isoforms of SigR, σ^R^ and σ^R′^, are produced from the two promoters of *sigR* gene, *sigR*p1 and *sigR*p2, respectively. Under cytoplasmic reducing environment, the reduced RsrA binds SigR (primarily the abundant σ^R^), inhibiting SigR-directed transcription. Upon oxidative stress by thiol-oxidants such as diamide, di-sulfide bonds are formed in RsrA, and SigR is released from sequestration. The released SigR directs transcription of more than 100 genes (SigR regulon) that includes its own gene (from the upstream *sigR*p2 promoter). In contrast to σ^R^ that is very stable, σ^R′^ with 55 more N-terminal amino acids is very unstable. (**B**) Difference between induction by thiol oxidant diamide and antibiotic tetracycline. The wild-type cells were sampled at 0, 30, 60 and 120 min after treatment with tetracycline (2 μg/ml, or 4.16 μM) or diamide (0.5 mM) for S1 nuclease mapping of *sigR*-specific RNAs. The rRNA in each RNA sample were resolved in parallel. Results from three independent experiments were quantified to present values for average fold induction with standard error of the mean (s.e.m.).

While performing hygromycin-chase experiment to measure the half-life of σ^R^ and σ^R′^ proteins, we previously observed an increase in transcripts from the *sigR*p1 promoter^29^. This observation was unexpected since we used to regard the *sigR*p1 promoter as constitutive. We examined the effect of other antibiotics and compared it with that of thiol oxidant diamide. Figure 1B shows the induction profile of *sigR*p1 and *sigR*p2 transcripts after treatment with tetracycline (2 μg/ml) or diamide (0.5 mM) for up to 2 h. Similarly to hygromycin, *sigR*p1 transcripts increased significantly by about 10-fold in response to tetracycline, in a prolonged fashion. This contrasts with the transient induction of *sigR*p2 transcripts by diamide as previously observed^19^,^26^. The antibiotic induction of *sigR*p1 transcription does not seem to be mediated by SigR itself, unlike *sigR*p2 transcription, since the induction occurred even in the Δ*sigR* mutant (MK1), where all transcriptions from the *sigR*p2 promoter disappeared (Fig. S1).

**Figure 2:**
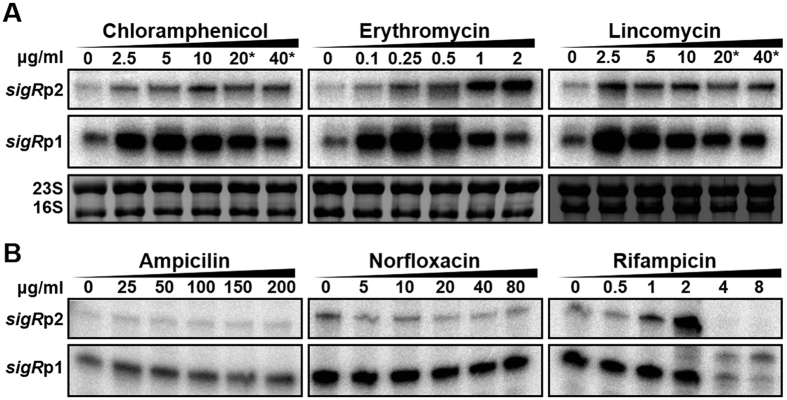
Translation-inhibiting antibiotics induce *sigR*p1 transcription. (**A**) Effect of antibiotics that target translation. *S. coelicolor* cells were sampled at 30 min after treatments with chloramphenicol (0 to 40 μg/ml), erythromycin (0 to 2 μg/ml), or lincomycin (0 to 40 μg/ml). S1 nuclease protection assay for *sigR*-specific transcripts were done. The rRNA in each sample were presented as a control. The asterisk (*) denotes inhibitory concentration of the antibiotics (see Fig. S1). (**B**) Effect of antibiotics that target other cellular processes; cell wall synthesis (ampicillin from 0 to 200 μg/ml), DNA replication (norfloxacin from 0 to 80 μg/ml), and transcription (rifampicin from 0 to 8 μg/ml). The *sigR*-specific RNA analysis was done as in panel (**A**).

To investigate the induction of *sigR* mRNAs by antibiotics in further detail, we explored diverse antibiotics with different chemical structures and targets. Following 30 min treatments at varying concentrations, the *sigR* transcripts were monitored by S1 mapping. The results demonstrated that translation-inhibiting antibiotics such as chloramphenicol, erythromycin, and lincomycin all induced *sigR*p1 expression significantly (Fig. 2A). Fusidic acid and streptomycin also induced *sigR*p1 transcripts (data not shown). On the other hand, ampicillin, norfloxacin, and rifampicin that affects cell wall, DNA replication, and transcription, respectively, failed to increase transcripts from *sigR*p1 (Fig. 2B). Rifampicin induced *sigR*p2 expression at 2 μg/ml, as observed previously in a different *S. coelicolor* strain M600^37^. Thus, the *sigR*p1 expression is induced specifically by translation-inhibiting antibiotics. Determination of growth inhibitory concentrations for treated antibiotics (Fig. S1) indicated that the *sigR*p1 induction occurred at sub-inhibitory concentrations.

### Antibiotic treatment increases σ^R^ protein and steadily induces target gene expression

**Figure 3:**
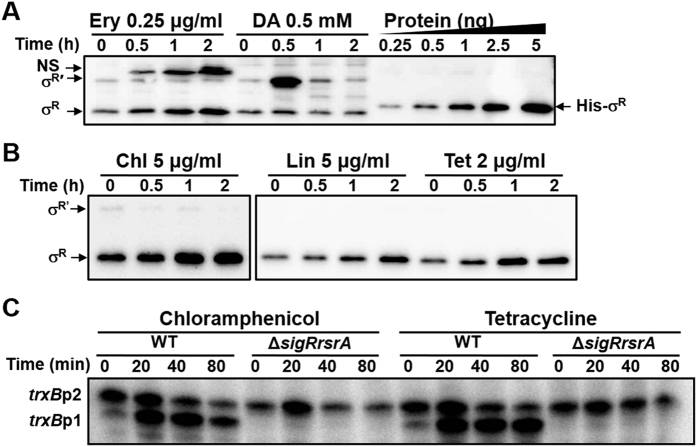
Steady increase in σ^R^ protein by antibiotic treatments and prolonged induction of its target promoter (*trxB*p1). (**A**) Steady vs. transient increase in SigR proteins by antibiotic or thiol oxidant. *S. coelicolor* cells were treated with either erythromycin (0.25 μg/ml) or diamide (0.5 mM) for up to 2 h, followed by western blot analysis with antibody against SigR. The positions of σ^R^ and σ^R′^ were marked by arrows. A non-specific band (NS) produced in erythromycin-treated samples was also indicated. Analytical western blotting of indicated amounts of purified SigR (His-σ^R^; from 0.25 to 5 ng) with anti-SigR antibody was done in parallel to quantify the amount of SigR-specific protein bands. (**B**) Western blot analyses of SigR proteins following treatments with chloramphenicol, lincomycin, and tetracycline for 2 h. (**C**) S1 mapping analysis of *trxB* transcripts. The wild-type (M145) and *ΔsigRrsrA* (MK1) cells were treated with chloramphenicol (17 μg/ml) or tetracycline (5 μg/ml) for up to 80 min, and analyzed for *trxB* transcripts. The *trxB*p1 promoter is under the control of SigR.

Whether the increase in *sigR*p1 transcripts leads to increased σ^R^ protein level in the presence of translation-inhibiting antibiotics was then examined. Analytical Western blot analysis with anti-SigR antibody revealed that erythromycin (0.25 μg/ml) increased the level of σ^R^, but not σ^R′^ protein, continuously for up to 2 h (Fig. 3A). This contrasts with the effect of thiol oxidant diamide which increased the amount of σ^R′^ transiently by about 12-fold, without affecting the level of σ^R^ (Fig. 3A). Parallel detection of known amounts of σ^R^ protein enabled the estimation that σ^R^ increased steadily by erythromycin to about 3-fold level at 2 h after treatment compared with the untreated level. The basal amounts of σ^R^ and σ^R′^ proteins under non-treated condition were estimated to be about 23 (1.82 μM) and 7 (0.56 μM) fmole/μg proteins in cell extracts, respectively, assuming equal immune-specificity of σ^R^ and σ^R′^ proteins to the antibody used. This corresponds to about 1.8 and 0.6 μM in the cell for σ^R^ and σ^R′^, respectively, assuming that about 43% of dry cell weight is from the protein, and that the wet cell weight is about 5.6 fold of the dry weight, and that cell density is 1^38^. Following erythromycin treatment, there appeared a non-specific band which is absent in other antibiotic-treated samples (NS in Fig. 3A). The source of this protein band is not certain, except that it is not the product of the *sigR* gene, since it is observed in the *ΔsigR* mutant after erythromycin treatment. Treatments with chloramphenicol, lincomycin, and tetracycline caused similar increase in σ^R^ without changing the amount of σ^R′^ (Fig. 3B). No increase in σ^R′^ by antibiotics in spite of some increase in *sigR*p2 transcripts could be due to the unstable nature of σ^R′ ^29.

We then examined the expression of a SigR-target gene *trxB* (SCO3890), which encodes thioredoxin reductase. Figure 3C shows that the SigR-dependent *trxB*p1 transcripts increased significantly by chloramphenicol and tetracycline treatments up to 80 min, consistent with the steady increase in σ^R^ protein. Therefore, we conclude that the translation-inhibiting antibiotics induce the production of stable σ^R^ protein, which subsequently induces its target gene expression in a prolonged fashion.

### Antibiotic induction of stable σ^R^ depends on WblC/WhiB7

**Figure 4:**
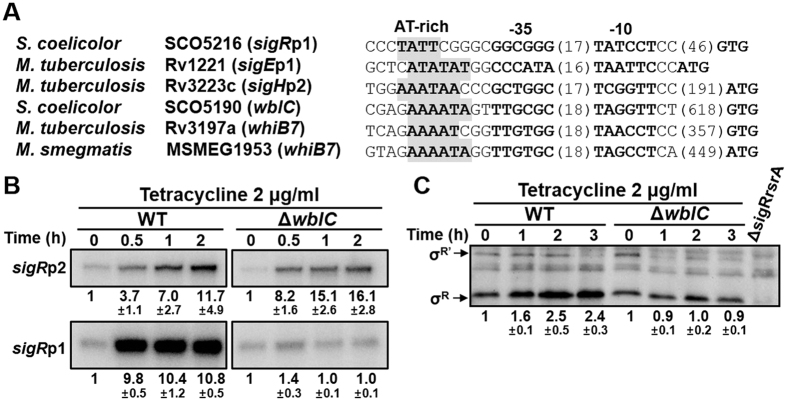
Antibiotic induction of *sigR*p1 transcription and σ^R^ production depends on WblC/WhiB7. (**A**) The presence of AT-rich sequence adjacent to the −35 element of promoters for *sigR*p1 in *S. coelicolor* and its homologous genes (*sigE* and *sigH*) in *M. tuberculosis*. The AT-rich sequences upstream of the *wblC* in *S. coelicolor*, and the *whiB7* promoters known to bind WhiB7 protein in mycobacteria, were also presented. (**B**) Antibiotic induction of *sigR*p1 transcripts depends on WblC/WhiB7. S1 nuclease mapping was done, following treatment of the wild type and Δ*wblC* cells with 2 μg/ml tetracycline for up to 2 h. Results from three independent experiments were quantified to present average values for relative fold change and s.e.m. (**C**) Western blot analysis of SigR proteins from cells similarly treated as in panel (**B**). Results from three independent experiments were quantified.

To find clues to reveal mechanisms behind antibiotic induction of *sigR*p1, we scrutinized its flanking sequences. One prominent feature was a stretch of AT-rich sequence, which is not common in GC-rich actinomycetous genomes, located immediately upstream of the −35 region of the *sigR*p1 promoter (Fig. 4A). This sequence feature is present upstream of the *whiB7* promoter in *Mycobacterium* species, and has been proposed as the binding site of a WhiB-like (Wbl) protein WhiB7^36^,^39^. In *S. coelicolor*, WblC (SCO5190) is the orthologue of WhiB7 of *M. tuberculosis*, and the *wblC* gene also has a putative auto-regulatory WblC-binding signature similarly to the *whiB7* gene of *M. tuberculosis* (Fig. 4A). The *wblC* and *whiB7* mutants were reported to be hypersensitive to diverse antibiotics in *S. lividans, S. coelicolor*, and *M. tuberculosis*^12^,^34^. Inspection of the promoter region of *sigR*-homologous genes (*sigE* and *sigH*) in *M. tuberculosis* H37Rv also revealed the presence of putative WhiB7-binding sites immediately upstream of the promoters^40^ (Fig. 4A).

We investigated whether WblC is involved in inducing transcription from the *sigR*p1 promoter upon antibiotic treatment. The wild type and the Δ*wblC* mutant cells^34^ were treated with tetracycline for up to 3 h, and examined for *sigR*-specific transcripts and their protein products by S1 mapping and Western blot analyses, respectively. Results in Fig. 4B demonstrated that WblC is critically required for the antibiotic induction of *sigR*p1 transcription. The *sigR*p2 transcription, however, was induced by tetracycline regardless of the *wblC* mutation. Immunoblot analysis revealed that the σ^R^ protein produced from the *sigR*p1 transcripts did not increase in Δ*wblC* mutant, in contrast to the wild type, where σ^R^ protein increased about 2.5-fold during the 2 to 3 h treatments with tetracycline (Fig. 4C). These results clearly show that the increase in stable σ^R^ after antibiotic treatment depends almost entirely on WblC/WhiB7.

### Antibiotics increase the amount and the binding of WblC to *sigRp1* promoter *in vivo*

**Figure 5:**
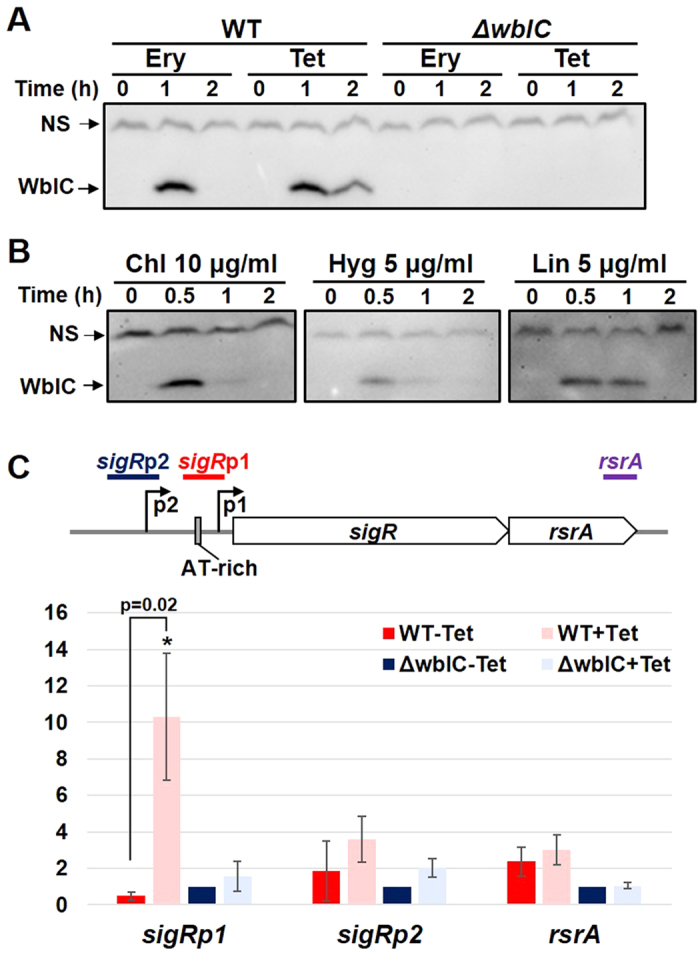
Increase in the amount of WblC protein and its binding to the *sigR*p1 promoter *in vivo* upon antibiotic treatments. (**A**) Western blot analysis of WblC. The wild-type and Δ*wblC* cells were sampled after antibiotic treatments; Ery for 0.25 μg/ml erythromycin, and Tet for 2 μg/ml tetracycline. The WblC-specific band was detected slightly below the 15 kDa marker, coinciding with its predicted size (13.2 kDa). NS denotes non-specific band. (**B**) Western blot analysis of WblC in wild type cells treated with other translation-inhibiting antibiotics; Chl for 10 μg/ml chloramphenicol, Hyg for 5 μg/ml hygromycin, and Lin for 5 μg/ml lincomycin. (**C**) Chromatin immunoprecipitation with anti-WblC polyclonal antibody followed by q-PCR with gene-specific primer sets for *sigR*p1, *sigR*p2, and *rsrA* genes. The wild-type and ΔwblC cells were processed for immunoprecipitation after 1 h treatment with or without 2 μg/ml tetracycline. The enrichment of each region was estimated by quantitative real-time PCR. The relative average fold with s.e.m. were presented (y-axis), by taking the value for untreated ΔwblC sample as 1. The asterisk (*) denotes p ≤ 0.05 by Student’s t-test (n = 3).

We then investigated how WblC is involved in antibiotic induction of *sigR*p1 or σ^R^. For this purpose, polyclonal antibodies against WblC were raised in rabbits, and used to monitor WblC in cells treated with antibiotics. Figure 5A shows that the amount of WblC dramatically increased within an hour of erythromycin or tetracycline treatments. The WblC level decreased within 2 h of antibiotic treatment. The decrease at 2 h is more pronounced in erythromycin than tetracycline treated samples. With some slight differences in induction and shut-off kinetics, WblC was induced by other antibiotics such as hygromycin, chloramphenicol, and lincomycin to a maximal level within an hour, and then returned to the basal level within 2 or 3 h (Fig. 5B).

Whether WblC binds directly to the *sigRp1* promoter region *in vivo* was determined by chromatin immunoprecipitation (ChIP) analysis. The wild type and the *ΔwblC* mutant cells were treated with tetracycline (2 μg/ml) for 1 h, followed by fixation, cell lysis, DNA shearing, and immunoprecipitation with anti-WblC antibody as described in Materials and Methods. The amount of *sigR*p1 promoter DNA in the precipitate was estimated by quantitative real-time PCR (qRT-PCR), along with probe sets for the upstream *sigR*p2 or downstream *rsrA* regions. Figure 5C demonstrates that tetracycline increased WblC binding to the *sigR*p1 promoter region (from −84 to +7 nucleotide position, relative to the transcription start site of *sigR*p1) by more than 10-fold in the wild type cell, whereas no increased binding was observed in the *ΔwblC* mutant. In comparison, no significant binding of WblC to the *sigR*p2 or *rsrA* regions was observed following tetracycline treatments. Therefore, we can conclude that the antibiotic treatments increase the amount of WblC, which specifically binds to the *sigR*p1 promoter region and mediates increased expression of σ^R^.

### SigR confers resistance to translation-inhibiting antibiotics

**Figure 6:**
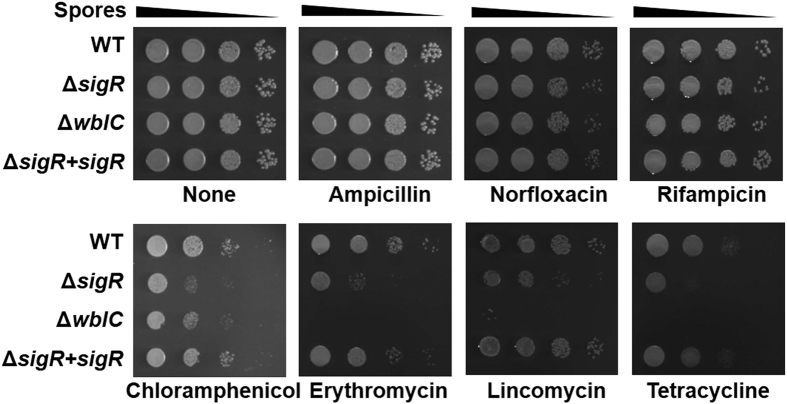
SigR confers resistance to translation-inhibiting antibiotics. An equal number of spores of the wild-type*, ΔsigR*, *ΔwblC*, and *ΔsigR*+*sigR* complemented strains were serially diluted by 10-fold and spotted on NA plates with or without antibiotics. Concentrations of antibiotics in the plates were 20 μg/ml ampicillin, 1 μg/ml norfloxacin, 2 μg/ml rifampicin, 10 μg/ml chloramphenicol, 2 μg/ml erythromycin, 10 μg/ml lincomycin, or 2 μg/ml tetracycline. Plates were incubated for 40 to 72 h.

On the basis of induction by antibiotics, we hypothesized that the *sigR* gene functions in conferring resistance to antibiotics in *S. coelicolor*. So far, the revealed phenotypes of *ΔsigR* mutant are the sensitivity to thiol oxidant diamide^19^, sensitivity to electrophiles (Park JH, unpublished), and increased protein aggregation in cell extracts that reflects decreased protein quality control^41^. To assess antibiotic sensitivity, we spotted an equal number of spores from the wild type, *ΔsigR, ΔwblC*, and *ΔsigR* complemented with the chromosomally integrated *sigR* gene, on plates containing various antibiotics. Figure 6 shows that the *ΔsigR* and *ΔwblC* mutations do not cause sensitivity toward non-inducing antibiotics such as ampicillin, norfloxacin, or rifampicin. However, as predicted, the *ΔsigR* mutant was more susceptible to inducing antibiotics such as chloramphenicol, erythromycin, lincomycin, and tetracycline. The sensitivity was restored to the wild type level by complementation with the wild type *sigR* gene. The *ΔwblC* was more susceptible than the *ΔsigR* mutant to the inducing antibiotics except chloramphenicol. These results demonstrate that the *sigR* gene does play a critical role in ensuring cell viability in the presence of translation-inhibiting antibiotics.

### Induction of *sigR*-homologous genes (*sigE* and *sigH*) by antibiotics in *M. tuberculosis*

**Figure 7:**
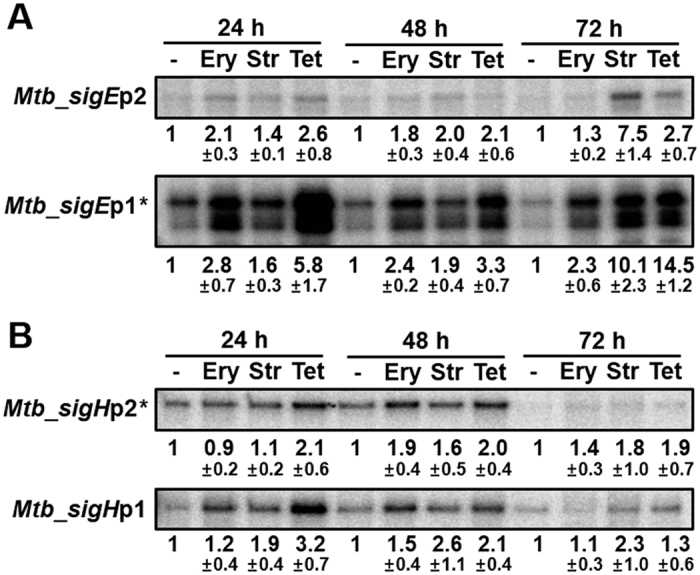
Induction of *sigR*-homologous gene (*sigE* and *sigH*) expression by antibiotics in *M. tuberculosis (Mtb).* Transcripts from the *sigE* panel (**A**) and *sigH* panel (**B**) genes of *Mtb* H37Rv strain were analyzed by S1 mapping. *Mtb* RNAs were obtained from cells grown in Middlebrooks 7H9 broth, and either non-treated or treated with 1 μg/ml of antibiotics for 24, 48, and 72 h: Ery, erythromycin; Str, streptomycin; Tet, tetracycline. Results from more than four independent experiments (n = 4 for *sigE* and n = 6 for *sigH*) were quantified to estimate changes in the level of transcripts, taking the level of untreated sample as 1. The average fold changes with s.e.m. were presented for transcripts from the p1 (downstream) and p2 (upstream) promoters of *sigE* and *sigH* genes. The *sigE*p1 and *sigH*p2 promoters (marked with *) contain putative WhiB7 binding motifs.

*M. tuberculosis* (Mtb) has two close homologs of SigR from *S. coelicolor* (ScoSigR); SigE (Rv1221; MtbSigE) and SigH (Rv3223c; MtbSigH) with 37% and 72% identity, respectively. SigH is known to regulate the thioredoxin system and heat shock proteins upon oxidative and heat stresses^42^,^43^. SigE plays a role in response to oxidative and cell envelop stresses^44^. The presence of predicted WblC binding sites in the promoter regions of *sigE* and *sigH* (Fig. 4A) led us to examine the expression of these genes in Mtb upon antibiotic treatments. We treated Mtb H37Rv cells with 1 μg/ml each of erythromycin, streptomycin, or tetracycline for up to 3 days. Figure 7 demonstrates the results of S1 nuclease mapping of transcripts from the *sigE* (panel A) and *sigH* (panel B) genes. For Mtb_*sigE* gene, we detected transcripts from the two promoters (transcription start sites) as have been reported^40^. The *sigE*p1 promoter contains the WhiB7-binding motif and produces leaderless mRNA (Fig. 4A). The upstream promoter *sigE*p2 does not have WhiB7-binding motif but contains the promoter sequence feature recognizable by MtbSigE or MtbSigH^45^. We found that the *sigE*p1 transcripts increased significantly by all three antibiotics (Fig. 7A). The *sigE*p2 transcripts increased also by antibiotic treatments, but by less pronounced fold of induction. For Mtb_*sigH* gene, we detected transcripts from two promoters; one from the downstream *sigH*p1 as reported previously^40^ and the other from the upstream *sigH*p2 recognizable by MtbSigH^42^,^46^. A WhiB7-binding motif is present in the *sigH*p2 promoter (Fig. 4A). Results in Fig. 7B show that both *sigH*p1 and *sigH*p2 transcripts increased by antibiotics, even though not as much as the *sigE* transcripts. Based on these observations, we can predict that similar pathways of upregulating SigR-like sigma factors by antibiotics are present in *M. tuberculosis*, and MtbSigE may play a more significant role in orchestrating response against translational blocking antibiotics.

## Discussion

In this work, we demonstrated that the *sigR* gene expression is induced by translation-inhibiting antibiotics to produce a stable isoform of SigR, σ^R^, which elevates its target gene expression for a prolonged period, in contrast to a transient induction of σ^R′^ by thiol-oxidative stresses. We also found that the *sigR* gene confers resistance to these inducing antibiotics. Previously, we identified 108 direct target genes of SigR by using ChIP-chip analysis^30^. Since the ChIP experiment was done after diamide treatment for 30 min, when the majority of the *sigR* gene product was σ^R′^ (more than 80% of the total SigR; Fig. 3A), the SigR regulon we determined reflects primarily the promoters preferentially bound by σ^R′^. Since σ^R′^ differs from σ^R′^ only by the N-terminal 55 amino acids, which may not affect promoter recognition, we consider the σ^R′^-bound genes may not differ from σ^R^-binding genes. Quite a number of SigR-target genes encode functions for thiol redox homeostasis, proteolysis, and ribosome modulation^30^,^41^.

Treatment with translation-inhibiting antibiotics will not only slow down the synthesis of new proteins, but also result in misfolded protein products due to mistranslation or protein truncation^47^,^48^. Stalled ribosomes uncoupled with transcription can cause mRNA cleavage, resulting in ribosome stuck at non-stop mRNA, which produces non-functional truncated protein upon ribosome rescue^49^,^50^. Therefore, the cellular damages caused by thiol-disturbing oxidative stress can overlap with those by translation-inhibiting antibiotics to quite an extent. In light of this, the functions of predicted ribosome-associated proteins of SigR regulon such as tmRNA (*ssrA*), RelA, HflX, peptide-releasing factor PrfA, EngA, and ObgE need be further investigated^30^.

Then, why is prolonged induction of SigR required to cope with antibiotics, whereas transient induction is sufficient to cope with oxidative stress? Our results implicate that *S. coelicolor* takes longer time to overcome antibiotic stress than thiol-oxidative stress. Thiol oxidants and electrophiles that elicit thiol-oxidative stress are efficiently removed in *Streptomyces* by mycothiol a functional equivalent of glutathione in actinomycetes^25^. Increased production and recycle of mycothiol, along with increased thiol-reducing systems, after thiol-oxidative stress will efficiently remove chemical stressors and return the thiol redox environment back to normal in a relatively short period of time. On the contrary, the antibiotics that bind to the ribosome is harder to be cleared from the cell, affecting cell physiology for longer period of time^51^. This may necessitate the utilization of stable regulator, such as stable σ^R^, that can carry out the response for prolonged period of time.

We observed that the antibiotics that induced *sigR*p1 transcription also induced *sigR*p2 transcription, even though to a lesser extent (Figs 1B,2A and 4B). The antibiotic induction of *sigR*p2 almost entirely depends on SigR, since no *sigR*p2 transcripts were observed in Δ*sigR* mutant (Fig. S1). Part of the reason that *sigR*p2 is induced by antibiotics is due to the secondary effect of increased σ^R^ that recognizes *sigR*p2. The results in Fig. 4B, which show that the *sigR*p2 is still induced by tetracycline in the Δ*wblC* mutant is not easy to explain. In the absence of WblC, no increase in σ^R^ is observed, and therefore, the *sigR*p2 induction is likely to occur via the pathway of inactivating RsrA (Fig. 1A). It can be speculated that somehow the intracellular environment of Δ*wblC* is more oxidized than the wild type following antibiotic treatment.

The more interesting question is how the production WblC protein is drastically elevated in the presence of translation-inhibiting antibiotics. The *wblC/whiB7* mRNA contains unusually long 5′ UTR with possible ORF for a small protein. This feature appears conserved across actinomycetes^52^, and may play some role in elevating WblC expression upon slowing down translation. There is also a possibility that the *wblC* gene expression partly depends on SigR, as predicted from the presence of SigR-dependent promoter sequence upstream of the *wblC* gene. The finding that the extent of antibiotic induction of the *sigR*p1 transcription in the *ΔsigR* mutant reduced to about half of the wild type level supports this idea (Fig. S1). Further studies are in need to unravel the underlying mechanism.

## Materials and Methods

### Strains, plasmids, and growth conditions

Spores of *S. coelicolor* A3(2) strain M145, *ΔsigRrsrA* disruptant (MK1)^29^ and *ΔwblC* disruptant^34^ were inoculated in YEME liquid medium containing 5 mM MgCl_2_•6H_2_O and 10% sucrose, and were grown at 30 °C^53^. NA plates (0.8% nutrient broth, 2% agar powder) were used for spotting analysis. *E. coli* was grown in LB broth. The pSET152H plasmid and *E. coli* ET12567, a non-methylating strain containing pUZ8002 for donor functions, were used for complementation as recommended^54^. *E. coli* DE3/gold strain and pET15b plasmid was used for WblC over-expression. *M. tuberculosis* H37Rv cells were grown at 37 °C in Middlebrooks 7H9 broth supplemented with 10% OADC and with or without antibiotics.

### Antibiotics and reagents

Antibiotics were obtained from Sigma-Aldrich and Duchefa biochemie. The solutions were prepared freshly before treatments.

### RNA preparation and S1 nuclease protection assay

*S. coelicolor* cells grown to OD_600_ of 0.3~0.4 in YEME were treated with various antibiotics or 0.5 mM diamide for 30~120 min. Harvested cells were disrupted by sonication in Kirby mix. RNA isolation and S1 nuclease protection assay were done as described previously^29^. For mycobacterial RNA preparation, harvested cells were re-suspended in TRIzol^®^ Reagent (Ambion, Life Technologies, Carlsbad, CA, USA), mixed with acid-washed 425~600 glass beads (Sigma-Aldrich G8772), and lysed using a mini-bead beater (BioSpec, Bartlesville, OK, USA). Following chloroform extraction and isopropanol precipitation, the RNA pellet was resolved in RNA-free water (Ambion, Life Technologies, Carlsbad, CA, USA). Mycobacterial RNA was analyzed by S1 nuclease protection assay as described previously^29^.

### Immuno-blot analysis

Cell lysates were obtained by sonication, and its protein concentration was determined as described previously^30^. To detect SigR, cell extracts containing 25 μg protein were diluted to the final concentration of 0.125 μg/μl with lysis buffer (200 μl) that contains 100 μg BSA to serve as a protein buffer. Aliquots of 8 μl containing 1 μg crude protein extract and 4 μg BSA were resolved on 15% SDS-PAGE. Immuno-detection was done by using polyclonal rabbit antibody against SigR and the anti-rabbit secondary antibody at 1:5000 dilution ratio, followed by ECL detection (Amersham Life Science). For detecting WblC, cell extracts containing 20 μg protein were resolved on 15% SDS-PAGE. Immuno-detection was done by using polyclonal rabbit antibody against WblC and the anti-rabbit secondary antibody at 1:10000 dilution ratio. All experimental protocols that involve animals were approved by and done in accordance with the guidelines by Seoul National University Institutional Animal Care and Use Committees (SNUIACUC).

### Spotting assay to monitor antibiotic sensitivity

NA plates containing various antibiotics (20 μg/ml ampicillin, 1 μg/ml norfloxacin, 2 μg/ml rifampicin, 10 μg/ml chloramphenicol, 2 μg/ml erythromycin, 10 μg/ml lincomycin, or 2 μg/ml tetracycline) were used to monitor sensitivity. An equal number of spores of wild type and mutant *S. coelicolor* strains were serially diluted by 10-fold and spotted on antibiotic-containing NA plates using a 48-pin replica plater (Sigma). The spotted plates were incubated at 30 °C for up to 3 days before taking photos.

### Chromatin immuno-precipitation

Exponentially grown cells (at OD_600_ of 0.3~0.4) were treated with 2 μg/ml tetracycline for 1 h, followed by fixation with 1% formaldehyde for 15 min. 125 mM glycine was subsequently added for 5 min at room temperature. Harvested cells were washed twice with cold TBS wash buffer (20 mM Tris-HCl, pH 7.5, 150 mM NaCl). To break cells and shear DNA, cells were sonicated in RIPA buffer (50 mM HEPES-KOH pH 7.5, 150 mM NaCl, 1 mM EDTA pH 8.0, 1% Triton X-100, 0.1% sodium deoxycholate, 0.1% SDS, 1 mM PMSF) with a sonicator (QSonica Q500) using a 3 mm tip at 30% maximum power, with 5 sec pulses for 15 times on ice. Following centrifugation at 13000 rpm and 4 °C for 10 min to clear the cell debris, 50 μl of each supernatant was set aside for input DNA control. To the cleared supernatant anti-WblC polyclonal rabbit antibody (5 μl) was added, and incubated at 4 °C for 1 h, with gentle mixing by rotation. Subsequently, 20 μl protein A/G beads (Santacruz) and 2 μg BSA were added and rotated overnight at 4 °C. The samples were centrifuged for 1 min at 4 °C and 3000 rpm and the pellets were washed once with low salt wash buffer (50 mM HEPES-KOH, pH 7.5, 150 mM NaCl, 1 mM EDTA, 1% Triton X-100, 0.1% sodium deoxycholate), once with high salt wash buffer (50 mM HEPES-KOH, pH 7.5, 500 mM NaCl, 1 mM EDTA, 1% Triton X-100, 0.1% sodium deoxycholate), once with LiCl wash buffer (10 mM Tris-HCl, pH 8.0, 250 mM LiCl, 1 mM EDTA, 1% NP-40, 1% sodium deoxycholate), and twice with TE buffer (10 mM Tris-HCl, pH 8.0, 1 mM EDTA). DNA was eluted by incubation in the elution buffer (10 mM Tris-HCl, pH 8.0, 250 mM NaCl, 1 mM EDTA, 1% SDS) at 65 °C for 30 min, followed by treatment with 5 μg proteinase K and 2 μg RNaseA for 1 h at 45 °C. NaCl was added to final concentration of 350 mM, and incubation continued at 65 °C overnight for reverse-crosslinking. DNA was purified by phenol-chloroform extraction. The amount of *sigR*p1, *sigR*p2, and *rsrA*-specific DNA was quantified by qPCR (Agilent Stratagene Mx3000P), using primer sets which encompass the *sigR*p1 promoter region (from −125 to −34 nt position, relative to the *sigR* start codon), *sigR*p2 promoter region (from −311 to −186 nt position, relative to the *sigR* start codon), and *rsrA* (from +920 to +991 nt position, relative to the *sigR* start codon).

## Additional Information

**How to cite this article**: Yoo, J.-S. *et al*. Induction of a stable sigma factor SigR by translation-inhibiting antibiotics confers resistance to antibiotics. *Sci. Rep.* **6**, 28628; doi: 10.1038/srep28628 (2016).

## Supplementary Material

Supplementary Information
