# Supplementary material for: Induction of a stable sigma factor SigR by translation-inhibiting antibiotics confers resistance to antibiotics
Source: Sci Rep. 2016 Jun 27;6:28628. doi: 10.1038/srep28628 (PMC4921905; doi:10.1038/srep28628)
Supplement: Supplementary Information [file srep28628-s1.pdf]

# Supplementary Information

# Induction of a stable sigma factor SigR by translation-inhibiting antibiotics confers resistance to antibiotics

Ji-Sun Yoo[1], Gyeong-Seok Oh[1], Sungweon Ryoo[2], and Jung-Hye Roe[1]

[1]Laboratory of Molecular Microbiology, School of Biological Sciences, and Institute of Microbiology, Seoul National University, Seoul 151-742, Korea

[2] Korean Institute of Tuberculosis, 168-5, Osongsaengmyeong 4-ro, Osong, Cheongwon-gun, Chungcheongbuk-do, 28158, Korea

Corresponding author; Jung-Hye Roe, jhroe@snu.ac.kr

Running title: antibiotic induction of an alternative sigma factor

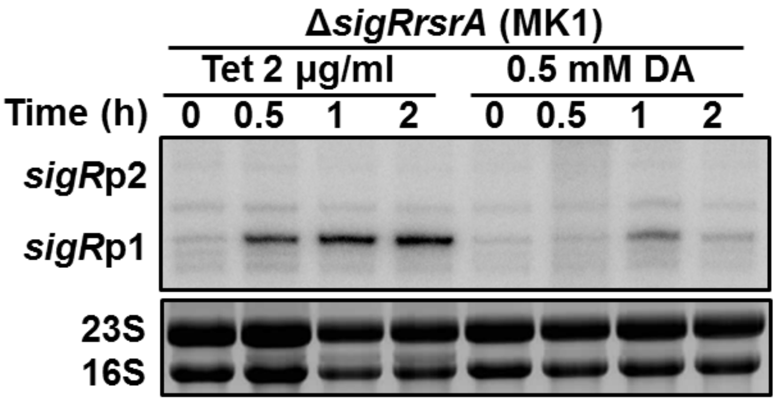


**Supplementary FIG S1. Persistent induction of *sigR*p1 transcripts by tetracycline in the absence of SigR.**

The *ΔsigRrsrA* (MK1) cells were sampled at 0, 30, 60 and 120 min after treatment with tetracycline (2 µg/ml) or diamide (0.5 mM) for S1 nuclease mapping. For each sample, 50 µg RNAs were analyzed for *sigR*-specific transcripts. Transcripts from *sigR*p2 promoter were below detection limit under all conditions. The *sigR*p1 transcripts were induced by tetracycline by about 4 to 6-fold during 0.5 to 2 h treatments. The rRNAs in each RNA sample were presented below.

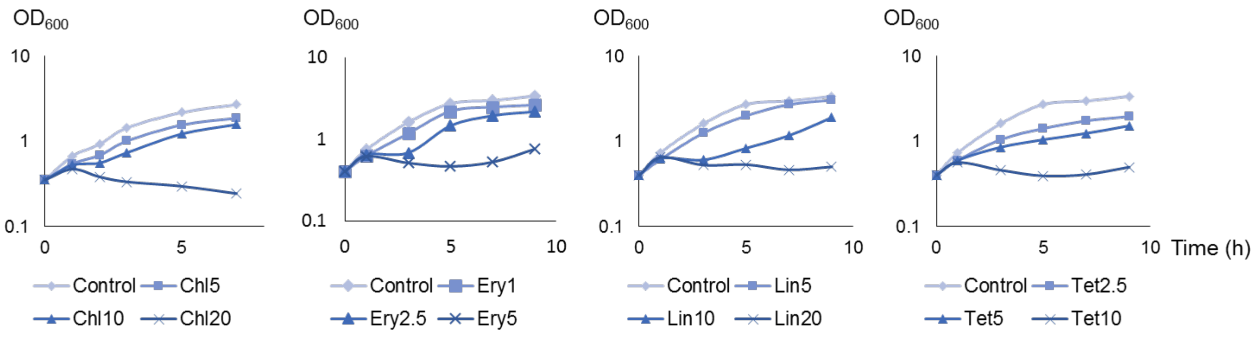


**Supplementary FIG S2 Inhibitory and sub-inhibitory concentration ranges of antibiotics used in this study.**

Growth of *S. coelicolor* M145 cells in YEME was monitored by $OD_{600}$. At $OD_{600}$ of ~0.4, varying concentrations of antibiotics were added to the culture medium, and the growth was monitored for up to 10 h. The amount of antibiotics treated was indicated in μg/ml for chloramphenicol (Chl), erythromycin (Ery), lincomycin (Lin), and tetracycline (Tet).
